# Synergistic regulation of molecular additives for stabilization of lithium-metal batteries

**DOI:** 10.1093/nsr/nwaf432

**Published:** 2025-10-13

**Authors:** Xinhai Yuan, Wenjing Ji, Lijun Fu, Hao Zhang

**Affiliations:** State Key Laboratory of Materials-Oriented Chemical Engineering, School of Energy Science and Engineering and School of Chemistry and Molecular Engineering and Nanjing Tech University, China; State Key Laboratory of Materials-Oriented Chemical Engineering, School of Energy Science and Engineering and School of Chemistry and Molecular Engineering and Nanjing Tech University, China; State Key Laboratory of Materials-Oriented Chemical Engineering, School of Energy Science and Engineering and School of Chemistry and Molecular Engineering and Nanjing Tech University, China; Department of Chemical Engineering, Massachusetts Institute of Technology, USA

Lithium metal is widely regarded as the ultimate anode for high-energy batteries due to its high specific capacity (3860 mAh g^−1^) and lowest electrochemical potential (−3.04 V vs. standard hydrogen electrode) [1]. Yet its practical use remains challenging. In conventional carbonate electrolytes, highly reactive lithium drives uncontrolled interfacial reactions that produce a fragile solid–electrolyte interphase (SEI) comprising inorganic (Li_2_CO_3_, Li_2_O) and organic (alkyl carbonate, alkoxide) species [2]. This SEI is mechanically weak and frequently fractures, causing continuous electrolyte consumption, dendritic growth and loss of active Li. On the cathode side, cathode electrolyte interphase (CEI) degradation couples with oxidative electrolyte decomposition, transition-metal dissolution and structural reconstruction, further accelerating capacity decay. Crosstalk between electrodes further propagates intermediates and side reactions, shortening battery life.

Electrolyte engineering—high-concentration and multi-solvent formulations, multi-salt strategies and functional additives—has aimed to steer interphase chemistry. Additives are attractive because they are cost-effective and manufacturing-compatible. Inorganic-rich interphases (e.g. LiF and Li_2_S) suppress parasitic reactions and promote uniform Li deposition [3,4]. However, carbonate solvents are inherently susceptible to nucleophilic attack; their decomposition products accumulate, hinder Li⁺ transport, increase polarization and exacerbate dendrite growth [5]. Thus, multifunctional additives that both enrich the inorganic species in the SEI/CEI and resist solvent decomposition are desired.

**Figure 1.**
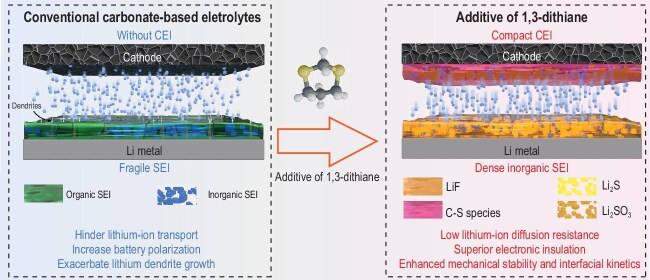
Interfacial regulation of lithium-metal batteries by 1,3-dithiane additive.

To address this challenge, Professor Wu and co-workers pioneered a molecularly adaptive strategy that, for the first time, achieves dual-electrode stabilization through a single molecular precursor. Specifically, they introduced 1,3-dithiane into conventional carbonate electrolytes (Fig. 1) [6]. This molecule contains two highly polarizable sulfur atoms that endow it with unique asymmetric redox properties: it undergoes preferential reduction at the lithium-metal anode to form LiF and Li_2_S, constructing a dense and ion-conductive SEI, while, at the high-voltage cathodes, it undergoes oxidation to yield Li_2_SO_3_ and organosulfur fragments, assembling into a compact CEI. Through this single-precursor redox bifunctionality, 1,3-dithiane enables the synchronous stabilization of both electrodes, mitigating crosstalk-driven

interfacial degradation in lithium-metal batteries (LMBs).

Electrochemical tests have validated this mechanism. With the addition of only 2 wt% of 1,3-dithiane, LiFePO_4_//Li full batteries retained 83.6% of their initial capacity after 3300 cycles at 1 C, with an average coulombic efficiency of 99.4%. Li//Li symmetric batteries cycled stably for >600 hours, which was nearly 4-fold longer than the control. The protective effect persisted under demanding conditions: ultra-thin lithium foil (50 μm), low N/P ratio (1.05), high cathode loading (1.9 mAh cm^−2^) and −10°C operation. In 1-Ah pouch batteries, the capacity retention exceeded 93% after 150 cycles, representing a significant improvement in cycle life, highlighting their scalability and practical potential.

Despite significant progress, several challenges remain on the path to industrialization, necessitating advances in both mechanistic understanding and engineering. One major bottleneck is oxidative stability at high voltage. Although 1,3-dithiane promotes the formation of a protective CEI, its stability at >4.3 V remains unproven under realistic conditions such as high areal loading, elevated temperature and lean electrolyte operation. Ni-rich cathodes not only suffer from lattice-oxygen release and phase transitions, but also trigger chain reactions of electrolyte oxidation that accelerate interfacial collapse. If the dithiane-derived CEI cannot tolerate these stressors, its protection will be short-lived. To enable reliable operation at ≥4.5 V, beyond simple molecular modification, the systematic structure–property mapping of dithiane derivatives, coupled with co-additive synergies, is needed to tailor both oxidation potential and film-forming ability. Advanced operando studies of interfacial redox chemistry will further clarify the degradation pathways and identify functional motifs for next-generation additives.

Another pressing issue is additive consumption and sustainability. As 1,3-dithiane is continuously consumed during interfacial reactions, its gradual depletion may weaken long-term protection, particularly under extended cycling and high-loading conditions. This raises key questions: Can the interphase remain stable once the precursor is depleted? What is the optimal dosage or replenishment strategy? To address these issues, quantitative consumption kinetics studies and electrolyte optimization are essential for guiding practical applications. Furthermore, controlled-release designs could sustain interphase protection over long lifetimes [7].

In addition, the mechanistic understanding of interphase evolution remains insufficient. Conventional *ex situ* analyses capture only endpoint states, ignoring transient species and dynamic structural transformations that critically govern long-term performance. Given the dependence of SEI/CEI formation on cycling history and time evolution, advanced operando tools, such as cryogenic transmission electron microscopy, synchrotron spectroscopy and time-resolved mass spectrometry, are indispensable for revealing nanoscale morphological changes, electronic structure variations and byproduct evolution. These insights provide a more robust foundation for rational additive design and predictive modeling, thereby bridging the gap between empirical observations and fundamental understanding.

Manufacturing compatibility represents the ‘last mile’ challenge. Additives must simultaneously balance viscosity, ionic conductivity and thermal stability to ensure processability during electrode winding, electrolyte infiltration and packaging. Moreover, uniform distribution across thousands of batteries and reliable performance in roll-to-roll production require stringent quality-control and life-cycle assessment. Equally important is the development of scalable synthesis and purification routes for additives to ensure consistency and reduce cost. Without these measures, laboratory success may not translate into industrial viability.

From an application standpoint, the key advantage of this strategy lies in achieving dual-electrode stabilization with a single molecular precursor, avoiding the complexity and cost of electrode-specific modifications. More importantly, 1,3-dithiane exemplifies the broader potential of molecular additive-enabled synergistic regulation for LMBs. Its simplicity, compatibility and scalability underscore its promise to extend lifespans, enhance safety and reduce manufacturing barriers, ultimately paving the way toward the practical commercialization of high-energy-density LMBs.

Molecular additive-based synergistic regulation offers a novel route to improving the interfacial stability of LMBs. As a representative example, 1,3-dithiane, with bifunctional redox behavior, stabilizes both electrodes and improves cycling stability and safety. Future efforts will focus on structural optimization, durability enhancement and process integration to accelerate the transition of this strategy from laboratory concepts to industrial applications, to support the next generation of safe and long-life energy-storage technologies.
